# Reduction of Breakdown Pressure by Filter Cake Removal Using Thermochemical Fluids and Solvents: Experimental and Numerical Studies

**DOI:** 10.3390/molecules26154407

**Published:** 2021-07-21

**Authors:** Zeeshan Tariq, Murtada Saleh Aljawad, Mohamed Mahmoud, Olalekan Alade, Muhammad Shahzad Kamal, Ayman Al-Nakhli

**Affiliations:** 1 Department of Petroleum Engineering, King Fahd University of Petroleum & Minerals, Dhahran 31261, Saudi Arabia; g201406240@kfupm.edu.sa (Z.T.); olalekan.alade@kfupm.edu.sa (O.A.); shahzadmalik@kfupm.edu.sa (M.S.K.); 2 Saudi Aramco, Dhahran 31261, Saudi Arabia; ayman.nakhli@aramco.com

**Keywords:** filter cake formation, filter cake removal, breakdown pressure, thermochemical, modeling

## Abstract

The process of well cleanup involves the removal of an impermeable layer of filter cake from the face of the formation. The inefficient removal of the filter cake imposes difficulty on fracturing operations. Filter cake’s impermeable features increase the required pressure to fracture the formation. In this study, a novel method is introduced to reduce the required breakdown pressure to fracture the formation containing the water-based drilling fluid filter cake. The breakdown pressure was tested for five samples of similar properties using different solutions. A simulated borehole was drilled in the core samples. An impermeable filter cake using barite-weighted drilling fluid was built on the face of the drilled hole of each sample. The breakdown pressure for the virgin sample without damage (filter cake) was 6.9 MPa. The breakdown pressure increased to 26.7 MPa after the formation of an impermeable filter cake. Partial removal of filter cake by chelating agent reduced the breakdown pressure to 17.9 MPa. Complete dissolution of the filter cake with chelating agents resulted in the breakdown pressure approximately equivalent to the virgin rock breakdown pressure, i.e., 6.8 MPa. The combined thermochemical and chelating agent solution removed the filter cake and reduced the breakdown pressure to 3.8 MPa. Post-treatment analysis was carried out using nuclear magnetic resonance (NMR) and scratch test. NMR showed the pore size redistributions with good communication between different pores after the thermochemical removal of filter cake. At the same time, there was no communication between the different pores due to permeability impairment after filter cake formation. The diffusion coupling through NMR scans confirmed the higher interconnectivity between different pores systems after the combined thermochemical and chelating agent treatment. Compressive strength was measured from the scratch test, confirming that filter cake formation caused added strength to the rock that impacts the rock breakdown pressure. The average compressive strength of the original specimen was 44.5 MPa that increased to 73.5 MPa after the formation of filter cake. When the filter cake was partially removed, the strength was reduced to 61.7 MPa. Complete removal with chelating agents removed the extra strength that was added due to the filter cake presence. Thermochemical and chelating agents resulted in a significantly lower compressive strength of 25.3 MPa. A numerical model was created to observe the reduction in breakdown pressure due to the thermochemical treatment of the filter cake. The result presented in this study showed the engineering applications of thermochemical treatment for filter cake removal.

## 1. Introduction

In an overbalanced drilling operation, the filter cake is formed on the wellbore wall due to the difference in hydrostatic pressure between the drilling fluids and the reservoir fluid. This filter cake creates a very thin impermeable layer. From the drilling perspective, this layer is very useful as it mitigates the loss circulation of drilling fluids in the permeable geological formation, but from production and reservoir perspectives, if this thin layer is not removed, then it can restrict the flow and causes an additional pressure drop that ultimately can reduce the productivity of the well [1,2,3]. The additional pressure drop due to the invasion of the drilling fluid into the reservoir formation is termed skin. In addition to the skin damage, the drilling fluids are not compatible with the reservoir rocks and fluids. They could cause the swelling of clays, emulsions, wettability alteration of the formation near the wellbore, and scale formations. Therefore, during well completion, the first step taken is to remove this impermeable layer. The removal of the filter cake is challenging, exhaustive, costly, and time consuming. This expensive task becomes more challenging in the long horizontal wells because of the longer contact time of the drilling fluids with the reservoir sections. The impermeable filter cake layer imposes an additional resistance to fracturing, especially in extended reach horizontal wells. This layer adds more strength to the wellbore and will increase the breakdown pressure required to break the reservoir in tight formation as well as in permeable formations.

Breakdown pressure is the pressure required to create the fracture in the reservoir and allows the fracturing fluid to penetrate in the given formation [4,5,6,7,8]. Fractures are created by injecting a pressurized fluid that provides conductive paths between the formation matrix, natural fractures, and the wellbore [9,10]. Water-based fluid, which most times contain around 99.5% of water-sand mixture with 0.5% additives, forms the slurry that is injected to propagate the fracture after an initial volume of pad fluids are injected to initiate the fracture [11,12,13]. Based on the type of formation and its characteristics, multiple mixtures of fracturing fluids can improve the fracturing process’s effectiveness. Typical fracturing fluids used to fracture unconventional reservoirs are oil, water, polymers, linear gel, methanol, or a combination of water and methanol [14,15].

When drilling a horizontal well, the drilling fluid will have more contact and residency in the wellbore than in the vertical well because of the length of the horizontal section. This will impose more damage due to the formation of the filter cake at the face of the formation. The drilling fluid solids will invade the formation and create a positive skin in the near-wellbore area in addition to the impermeable filter cake [16,17,18,19]. Water-based drilling fluids are the common type of drilling fluids used to drill different well types due to their low environmental impact compared to oil-based drilling fluids. In some cases, in which the formation rock is very sensitive to water, oil-based drilling fluids prevent the formation damage and wellbore stability issues. Different types of oil-based drilling fluids are used to drill sensitive formations, such as invert-emulsion drilling fluid [20]. The filter cake removal in oil-based drilling fluids is different than that in the case of water-based because of the difference in the composition of the filter cake. Table 1 summarizes some of the scenarios for filter cake removal.

Barite is a commonly used weighting material in oil- and water-based drilling fluids to drill deep in oil and gas reservoirs. Barite has very low solubility in mineral and organic acids, compared to other weighting materials in drilling fluids. Barite also has moderate solubility in high pH chelating agents such as EDTA and DTPA [23]. The filter cake can be removed through either a single-stage or multi-stage process based on the computability of the remover fluid ingredients [23]. This study aims to investigate the removal of barite filter cake and its impact on the breakdown pressure of different rocks. In this work, thermochemical fluids (TCF) were used after simulated drilling operations to remove the barite-based filter cake completely. The new method can remove the barite-based filter cake and create microfractures and tiny cracks that can increase wells’ productivity and are instrumental in wells’ flowback period. This can expedite the operation of well cleanup.

## 2. Materials and Methods

### 2.1. Drilling Fluid Formulation

In this study, the typical field formulations were used to form the filter cake for the water-based drilling fluids. In water-based drilling fluid, 0.691 barrels (bbls) of the water was used. The drilling fluids additives were comprised of barite, sodium chloride (NaCl), potassium chloride (KCl), calcium carbonate (CaCO_3_), bentonite, Xanthan Gum (XC) polymer, and potassium hydroxide (KOH). The amount of drilling fluid additives was: barite was 352 lbs, NaCl 66 lbs, KCl 20 lbs, CaCO_3_ 5.0 lbs, bentonite 4.0 lbs, XC polymer 0.5 lbs, and KOH 0.5 lbs.

### 2.2. Core Preparation

**Figure 1:**
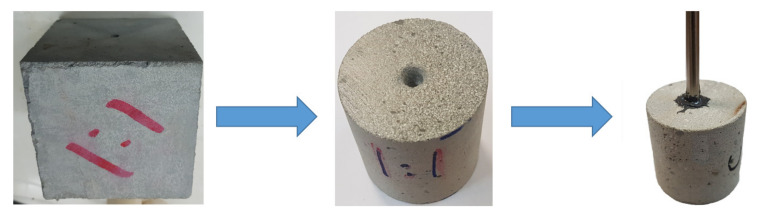
Illustration of the sample preparation.

Different cement cubicle blocks with a cement and sand ratio of 1:1 were prepared. The blocks were 101.6 mm long and 101.6 mm wide. The cylindrical core samples were taken out from the blocks. The dimensions of the cylindrical core samples were 50.8 mm in diameter and 50.8 mm in length. A synthetic borehole was created at the center of each specimen, which represents the wellbore with the dimension of 6 mm diameter and 19 mm depth. The cement samples were cast according to guidelines given in previous publications [33,34,35,36,37,38]. The illustration of sample preparation is given in Figure 1. The petrophysical and mechanical properties of cement samples are described in Table 2.

### 2.3. Thermochemical Fluids

To treat the water-based drilling fluid filter cake, thermochemical fluids were utilized. The thermochemical used in this study consisted of salts of nitrogen such as ammonium chloride $\left({\mathrm{NH}}_{4}\mathrm{Cl}\right)$ and sodium nitrite $\left({\mathrm{NaNO}}_{2}\right)$. The reaction between ${\mathrm{NH}}_{4}\mathrm{Cl}$ and ${\mathrm{NaNO}}_{2}$ is irreversible and highly exothermic that resulted in the in situ generations of nitrogen gas [39]. This reaction is normally considered a green reaction because it is mainly composed of salts [40,41]. Due to the high-pressure temperature generation, thermochemical fluids were successfully used in the oil and gas industry for several purposes such as wax removal [42], heavy oil production [43], breakdown pressure reduction [44,45,46], increasing stimulated reservoir volume [47], and enhanced oil recovery (EOR) applications [48]. Mahmoud [23] used thermochemical fluids along with chelating agents to remove the filter cake formed by barite-weighted drilling fluids in oil- and water-based types. He reported a removal efficiency of 80% and more in different cases. Ba Alawi et al. [49] used thermochemical fluids combined with hydrochloric acid to stimulate heterogeneous carbonate formations through the acid diversion mechanism that was enforced by TCF. They showed that TCF combined with HCl can stimulate large contrast in permeability. They conducted parallel core flooding experiments using high and low permeability carbonate rocks, and both rocks were stimulated efficiently due to the presence of TCF. Aljawad et al. [50] showed that TCF could generate very high pressure and temperature through fractured reservoirs. They showed that TCF could be combined with hydraulic fracture treatments to create a complex fracture network to enhance the well productivity.

The thermochemical reaction between ${\mathrm{NH}}_{4}\mathrm{Cl}$ and ${\mathrm{NaNO}}_{2}$ can be given by Equation (1) as follows:

(1) $${\mathrm{NH}}_{4}\mathrm{Cl}+{\mathrm{NaNO}}_{2}\to \left[\begin{array}{c} NH_{4}NO_{2}+NaCl.NH_{4}NO_{2}\\ Themolabile \end{array}\right]\to \mathrm{NaCl}+2H_{2}0+N_{2}\left(\mathrm{gas}\right)+\Delta H\left(\mathrm{heat}\right)$$

The reaction kinetics of the thermochemical fluids that were utilized in this study to remove the drilling fluid-based filter cake was extensively investigated, and the necessary reaction parameters such as specific heat capacity (*C*), enthalpy change $\left(\Delta H\right),$ and thermal conductivities $\left(\lambda \right)$ were determined. These reaction parameters are reported in Table 3.

**Figure 2:**
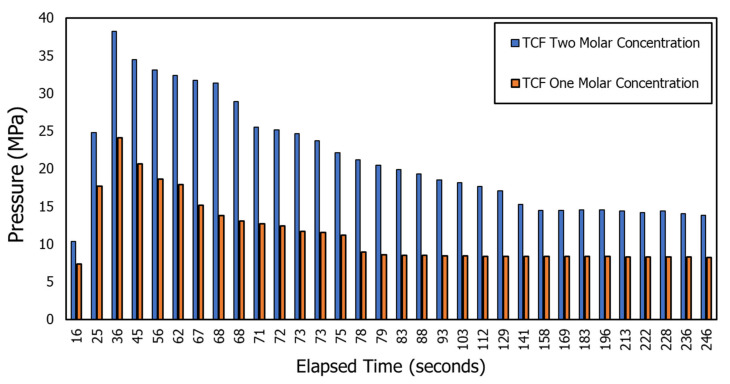
Effect of reactants molar concentration on pressure pulse generation due to thermochemical fluids.

Thermochemical fluids resulted in the generation of enormously high pressure. Figure 2 shows the plot of pressure generated by the reaction of one molar and two molar concentrations of ${\mathrm{NH}}_{4}\mathrm{Cl}$ and ${\mathrm{NaNO}}_{2}$ in an insulated high pressure and high temperature microreactor. Two molar concentrations of thermochemical fluids could generate a pressure of 38 MPa in 36 s, while one molar concentration of thermochemical fluids could generate a pressure of 24.13 MPa at the same time.

### 2.4. Chelating Agent

In this study, diethylenetriaminepentaacetic acid (DTPA) and glutamic acid (GLDA) chelating agents were used, along with the TCFs to remove the filter cake. The role of the chelating agent is to react and make a stable product. DTPA can dissolve the rock minerals. Through core-flooding experiments, Barri et al. [51] found that DTPA can create wormholes in the carbonate reservoir rocks. Recently in our previous study [52], we found that chelating agents can improve the fracture conductivity by many folds because of the rock dissolution. Based on previous experiences, DTPA and GLDA were used to dissolve filter cake. The reaction between TCFs occurs when a high temperature is reached. The fluids are pumped at the surface temperature and gain heat as they approach the wellbore downhole. The reaction will occur near the damaged zone, which usually takes 30 to 60 min to start. The study suggests adding chelating agents such as DTPA to the TCFs, which is a noncorrosive agent.

### 2.5. Experimental Setup

**Figure 3:**
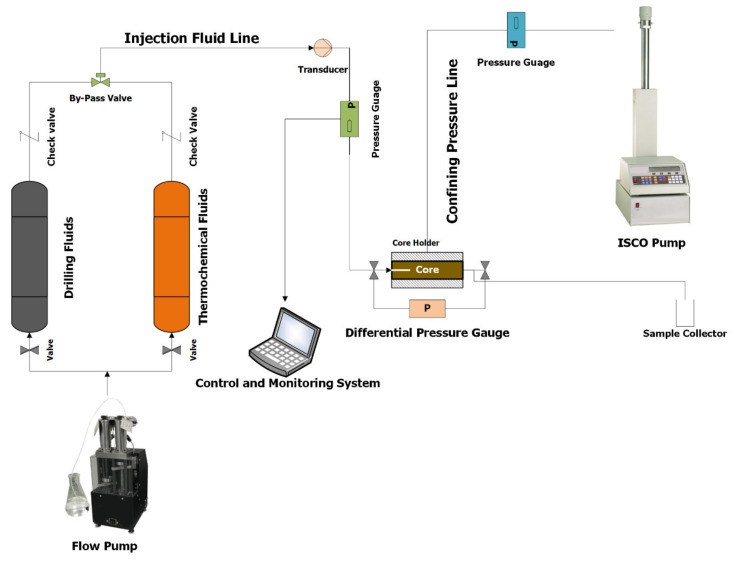
A schematic of filter cake and breakdown pressure setup.

The experimental setup comprised of a high-pressure injection pump, a 50.8 mm core holder, two-piston accumulators of volume one liter, a high-resolution data acquisition and monitoring system, high-pressure transducers, high-pressure valves, and high-pressure injection lines. Figure 3 shows the experimental process flow diagram used to form the filter cake. The same setup was used to dissolve/remove the filter cake and performing the hydraulic fracturing experiments.

The experimental study proceeded with the formation of the filter cake on the borehole wall of the samples, a typical example of the sample with a borehole is shown in Figure 1.

To create the filter cake, a drilling fluid was injected. To create and remove the filter cake, the breakdown pressure setup was used. The filter cake was created by applying a pressure difference of 200 psi at an ambient temperature condition. The filter cake is impermeable, which means it has zero permeability. The impermeable nature of the filter cake was tested by applying a constant pressure of 100 psi on the borehole of the cylindrical samples for 2 h. The pressure did not decline and remained constant over 2 h, confirming the presence of filter cake. After the generation of the filter cake, the same setup was used to remove the filter cake.

To remove the filter cake, an experimental plan was followed. Table 4 shows the experimental plan, the number of fracturing experiments, the type of fracturing fluids used to fracture the samples having the filter cake, and the type of characterization technique used after the fracturing experiment. A total of five fracturing experiments were conducted. Different chelating agents and thermochemical fluids were used.

**Figure 4:**
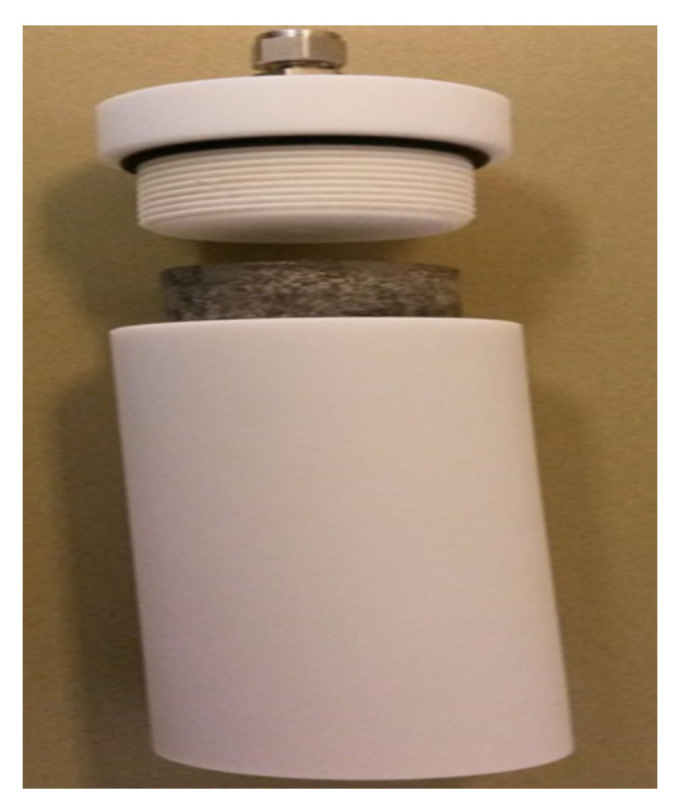
A schematic of the NMR core holder.

Figure 4 shows the NMR core holder used to scan the rocks before and after fracturing. The Teflon cell can carry a pressure up to 500 psi, the rocks were saturated with 3 wt% KCl brine at 1000 psi for 24 h before the NMR scans. A 2 MHz NMR core analyzer was used.

## 3. Results and Discussions

### 3.1. Breakdown Pressure Results

**Figure 5:**
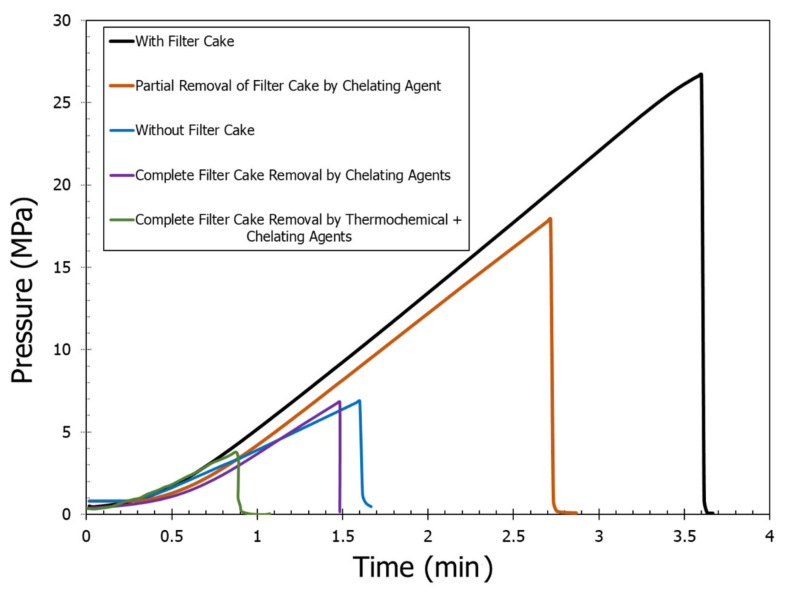
Pressure–time curves for different cases.

**Figure 6:**
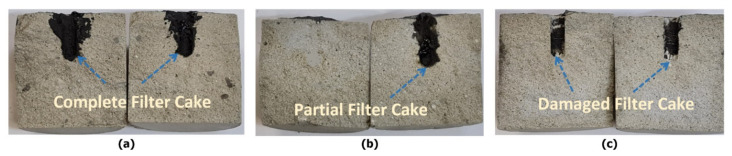
Cross-sectional views of the specimens treated with different fluids: (**a**) complete filter cake, (**b**) partial filter cake, and (**c**) damaged filter cake from combined thermochemical fluids and chelating agents.

A total of five breakdown pressure experiments were carried out on five different samples with and without having filter cake. When there was no filter cake, the original rock breakdown pressure with water was 6.88 MPa. The filter cake increased the required breakdown pressure from 6.88 MPa to 26.7 MPa. The 20 wt% DTPA chelating agent at 200 °F was used to partially remove the filter cake. Approximately, 45% of the filter cake was removed when soaked for six hours in a DTPA solution. The pH of DTPA was maintained at 11. The partial removal of filter cake resulted in a breakdown pressure of 17.93 MPa. The fourth experiment was carried out with the combination of different chelating agents. A mixture of 20 wt% DTPA chelating agent, 10 wt% GLDA, and 9 wt% K_2_CO_3_ was used at 200 °F temperature to completely dissolve the filter cake. The overall pH of the solution was 11. After the complete removal of the filter cake, a breakdown pressure of 6.82 MPa was observed. The fifth experiment was carried out with the combination of a chelating agent and thermochemical fluids. In this experiment, thermochemical fluids resulted in the generation of high pressure and high temperature. High pressure was generated due to the liberation of nitrogen gas, which resulted in the creation of micro and macro cracks in the sample. The high-temperature generation triggered the chelating agents. The combined effect of thermochemical fluids and chelating agents resulted in a significant reduction of the breakdown pressure to 3.78 MPa. Figure 5 shows the continuous injection pressure versus time curve for the different cases. The injection pressures were recorded till the rock fracture. The peak pressure corresponds to the breakdown pressure. Figure 6 shows the cross-sectional views of the different fractured samples.

**Figure 7:**
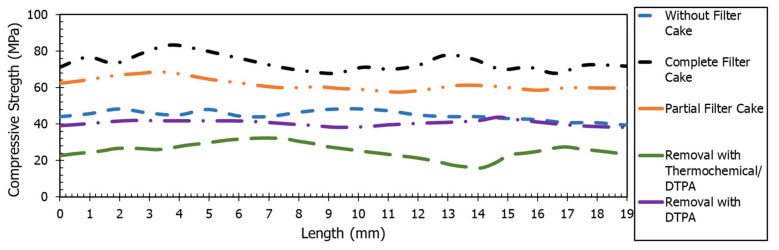
Continuous compressive strength profiles were recorded from the scratch test of the fractured rocks with different fracturing fluids. The strength measured on the cross section of the sample from top to the bottom along the borehole.

The compressive strength of the treated samples was measured from the scratch test. Scratch test is an indirect way to measure the continuous compressive strength. The strength was measured by creating a groove along the length of the sample on the cross section, from the top (0 mm depth) to the bottom (19 mm depth) along the borehole. The average compressive strength of the sample without any filter cake was 44.5 MPa that increased to 73.5 MPa after the creation of the complete filter cake. When the filter cake was removed partially, the strength was reduced to 61.7 MPa. Complete removal with chelating agents removed the extra strength that was added due to the filter cake presence, resulting in 40.4 MPa. Filter cake removal with the combined thermochemical and chelating agents resulted in a significantly lower compressive strength of 25.3 MPa. Figure 7 shows the variation in the continuous compressive strength profiles of the fractured rock samples for five different cases. Scratch test results are aligned with the breakdown pressure and confirmed that the filter cake formation caused added strength to the rock that impacts the rock breakdown pressure.

**Figure 8:**
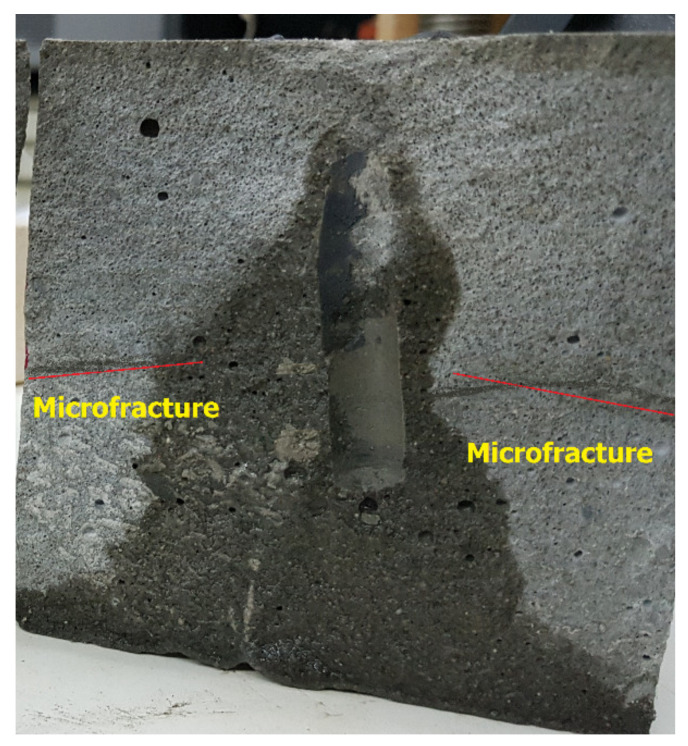
A cross-sectional view of the fractured sample showed the presence of microfractures (red line) due to the thermochemical treatment.

Figure 8 shows the cross-sectional view of the fractured 101.4 mm by 101.4 mm cubicle block. The picture was taken after the thermochemical reaction where the block was fractured. The microfractures were created as a result of the huge pressure generated from the thermochemical reaction. The thermochemical treatment for the filter cake created microfractures around the hole (wellbore), and these micro fractures reduced the required breakdown pressure compared to the native rock state.

### 3.2. NMR Results

**Figure 9:**
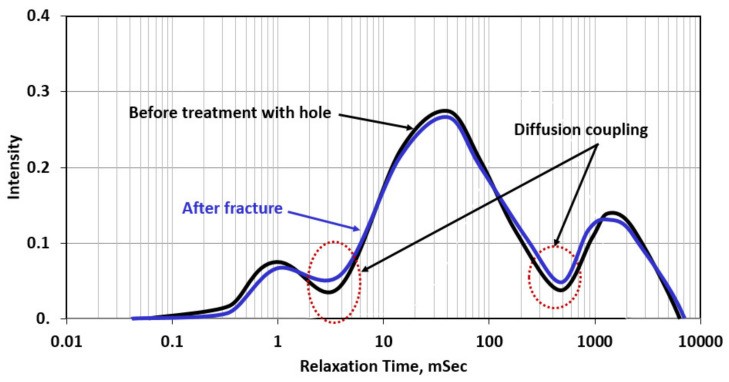
NMR scans for the rock samples without filter cake before and after fracturing (Experiment 1).

NMR scans were conducted for different cases by coring 38.1 mm diameter samples from the original cylindrical samples. Figure 9 shows the NMR scans for the rock sample without filter cake before and after fracturing with water. NMR showed that the rock sample has three pore systems that are well connected (through diffusion coupling). The largest pore system represents the drilled hole in the rock sample. After fracturing with water, NMR diffusion coupling showed a better connection between the micro- and mesopore system due to the creation of the fracture. The three peaks in the NMR scan represent micro-, meso-, and macropores in which the micro appears at relaxation time less than 2 msec and the meso between 3 and 300 msec, and the macro appeared at relaxation time greater than 400 msec.

**Figure 10:**
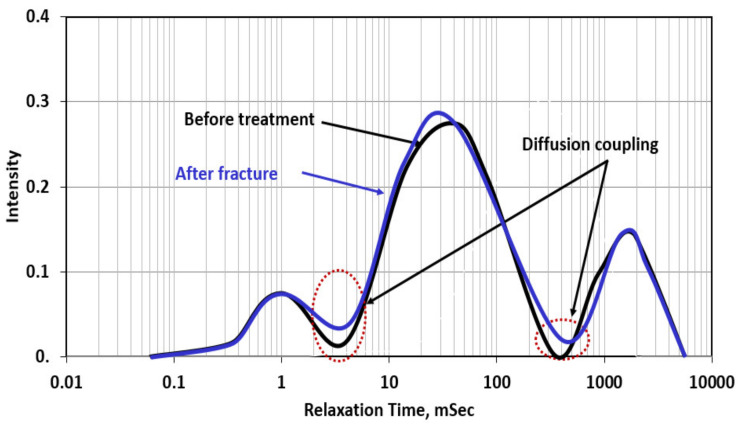
NMR scans for the rock samples with filter cake before and after fracturing (Experiment 2).

Figure 10 shows the NMR scans for the rock with the formed filter cake (barite weighed in water-based drilling fluid). The rock was scanned twice; the first one represents the rock with the filter cake (before treatment) in which the filter cake completely plugged the connections between the drilled hole and the rock, in addition to partial plugging for the connection between micro- and mesopores due to the invasion of the drilling fluid filtrate. After fracturing with water, in the presence of the filter cake, the created fracture enhanced the connectivity between the hole and the rock. Additionally, the connectivity between micro- and mesopores was enhanced due to the creation of the fracture. Compared to Figure 9, the filter cake reduced the diffusion coupling between different pore systems in the rocks and this is reflected by the decrease in the intensity of the diffusion coupling. Filter cake formation around the drilled hole reduced caused isolation between the hole and the rock, and as shown in Figure 10, there is complete isolation between the meso- and macropores system in which the intensity reached zero.

**Figure 11:**
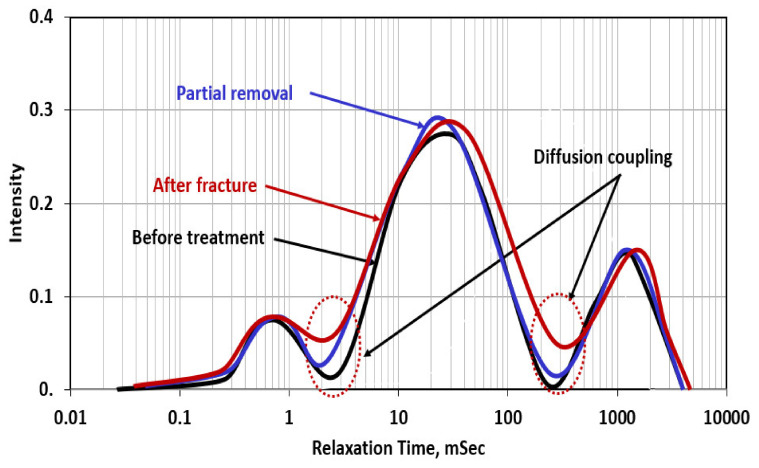
NMR scans for the rock samples with partial filter cake before and after fracturing (Experiment 3).

Figure 11 shows the NMR scans for the case of partial filter cake removal using a 20 wt% DTPA chelating agent at 200 °F. The removal efficiency was 40%, which means still 60% of the filter cake is covering the wall of the drilled hole. The NMR scan for the rock with the filter cake showed very similar behavior to that in the case of Figure 10 in which the filter cake completely plugged the connection between the hole (macropores) and the rest of the pore system. This plugging was confirmed by a zero-diffusion coupling between the hole and the rock, in which no communication between the two-pore systems, and the H^+^ protons cannot diffuse between the two-pore systems. This happened due to the zero permeability of the filter cake that is formed at the wall of the hole. The NMR scan shows that after the partial removal of the filter cake, the connectivity between the hole (macropores) and the rest of the rock system is partially restored, and the diffusion coupling between the different pore systems is partially restored, compared to before the filter cake removal. The created fracture enhanced the connectivity between the hole and the rest of the pore systems, as indicated by the increase in the diffusion coupling intensity between macro-/mesopore and meso-/micropore systems. Mahmoud [53] introduced the concept of the interconnectivity between the different pore systems in the rock using the diffusion coupling from NMR scans. In this case, the interconnectivity number is the value of the intensity of the diffusion coupling between the macro-/mesopore system to that of the meso-/micropore system. In Figure 11, the three cases interconnectivity number (ICN) can be presented as follows:

1. Before treatment, in which the filter cake completely plugged the connection between the hole and the pore systems in the rock, ICN = 0/0.025 = zero;
2. In the case of partial removal of the filter cake, ICN = 0.02/0.03 = 0.67;
3. After creating the fracture (with water) through the partially removed filter cake, ICN = 0.045/0.65 = 0.70.

**Figure 12:**
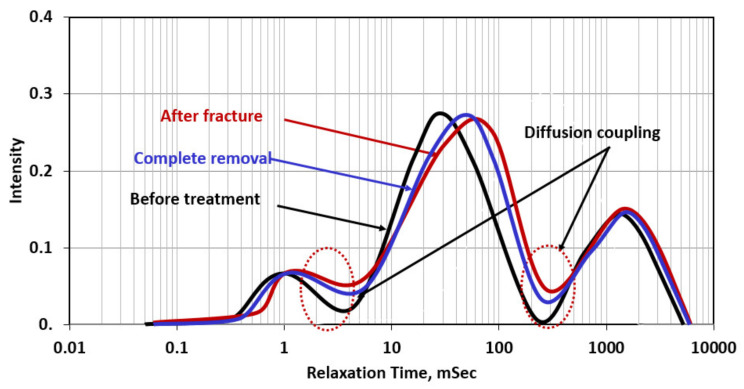
NMR scans for the rock samples with complete removal of the filter cake before and after fracturing (Experiment 4).

Figure 12 shows the NMR scans for the case of complete filter cake removal using 20 wt% DTPA chelating agent at pH 11 + 10 wt% GLDA at pH 11 + 9 wt% K_2_CO_3_ at 200 °F for 24 h. In this case, the filter cake caused very similar damage to the previous cases and completely plugged the interconnectivity between the hole and pore system in the rock. The complete removal of the filter cake (removal efficiency more than 90%) resulted in enhancement in the diffusion coupling between the different pore systems in the rock (macro/meso and meso/micro). The created fracture using water has further enhanced the connection and increased the intensity of the diffusion coupling. In applying the concept of the interconnectivity number in this case, the following are the results of ICN estimation for the three NMR scans:

1. Before treatment in which the filter cake completely plugged the connection and covered the wall of the hole, ICN = 0 /0.25 = zero;
2. Complete removal of the filter cake: in this case, the remover fluid removed both the filter cake and the polymer; the used formulation consists of GLDA chelating agent that can break the polymer coat and DTPA + K2CO3 to remove the barite. In this case, the ICN = 0.3/0.4 = 0.75;
3. After fracture (with water), the ICN = 0.45/0.60 = 0.75.

The creation of the fracture with water did not improve the ICN value after the complete removal of the filter cake, which indicates the inefficiency of using water to create a fracture in tight rocks.

**Figure 13:**
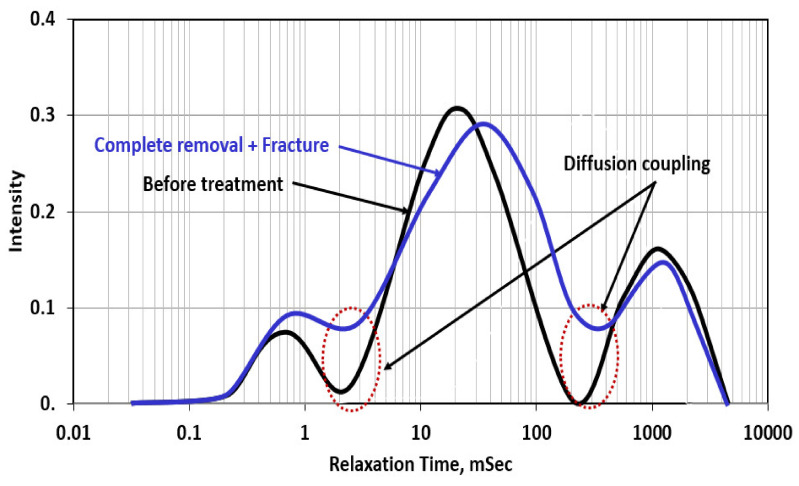
NMR scans for the rock samples with complete removal of the filter cake before and after fracturing (Experiment 5).

Figure 13 shows the NMR scans for the last case in which TCF combined with chelating agent and catalyst to remove the filter cake and create multiple fractures in the rock. The fluids used here are two thermochemical fluids (sodium nitrate + ammonium chloride); when reacting at the reservoir temperature, they will generate a very high-pressure pulse that will create major and microfractures. In addition, the reaction will generate a high temperature (an additional 100 °F), which will promote the reaction of the chelating agent and catalyst with the filter cake. The interconnectivity number calculations can be presented as follows:

1. Before treatment, in which the filter cake completely covered the hole, ICN = 0 / 0.02 = zero;
2. Complete removal + fracture; in this case, the filter cake was completely removed, and TCF created a fracture and microfractures around the hole, ICN = 0.075 / 0.08 = 0.94. The interconnectivity number, in this case, was the highest among all cases presented in this study due to the creation of microfractures (as shown in Figure 8), in addition to the major fracture. In addition, the complete removal of the filter cake obtained from the thermochemical treatment enhanced the communication between the different pore systems in the rock.

**Figure 14:**
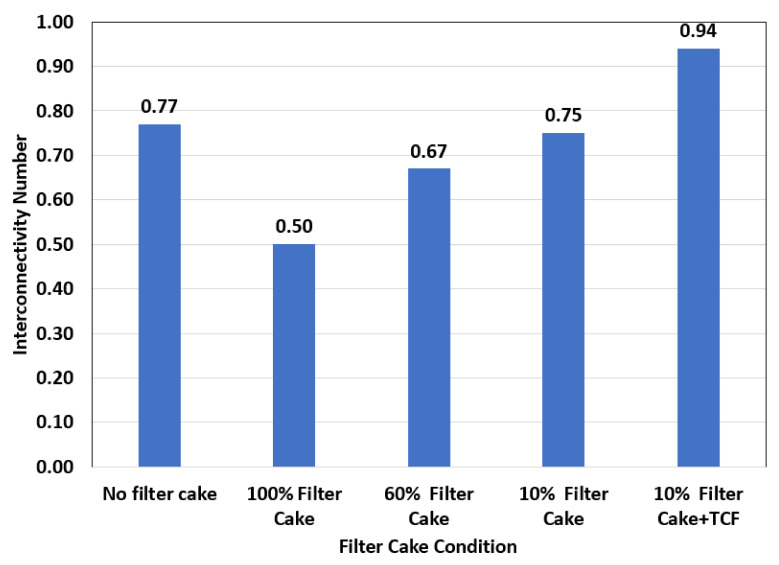
Relationship between filter cake condition and interconnectivity number (ICN).

Figure 14 shows the effect of filter cake conditions on the interconnectivity number. There is a strong relationship between the filter cake removal efficiency and the ICN and the effect of the type of remover. The ICN indicates the degree of connectivity between the different pore systems in the rock. The initial conditions of the rock without filter cake showed an ICN of 0.77, which shows good connectivity between the macro-/mesopores and meso-/micropores in the rock. The ICN was estimated for the samples after fracturing treatment with either water or TCF. Even fracturing could not restore the interpore connectivity due to the formation of the filter cake; the ICN value was 0.5 for the fractured rock with no filter cake removal. This elucidates the mandatory need for filter cake removal to restore the communication between the pore systems. The fractured rock after partial removal of the filter cake (removal efficiency 40%, 60% of the filter cake still exists) yielded an ICN of 0.67, which also indicates that partial removal of the filter cake will not provide favorable conditions for the flow and will not restore the communication between the pores ad the fracture and this will impact the well’s productivity. The complete removal of the filter cake (removal efficiency 90%, 10% of the filter cake still covering the wall of the hole) restored the pore connectivity after fracturing to almost the original value; the ICN, in this case, was 0.75, compared to 0.77 in the case of the fractured rock without filter cake. The application of thermochemical fluid (TCF) in addition to the filter cake remover (20 wt% DTPA + 10 wt% GLDA at pH 11 + 9 wt% K_2_CO_3_) yielded the highest ICN value after fracturing. The ICN, in this case, was 0.94, which is much higher, compared to all the reported cases, and higher compared to the original rocks without filter cake. This means that the addition of TCF enhanced the connectivity between the pore systems through the creation of the main fracture and microfractures around the whole.

**Figure 15:**
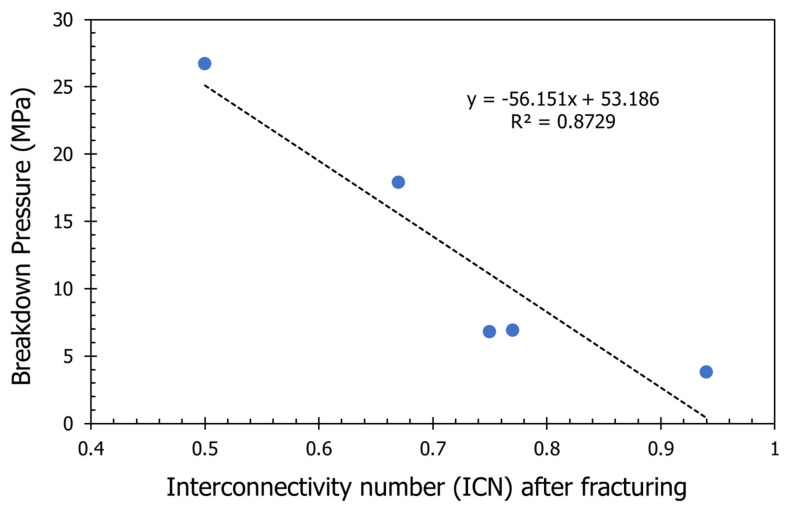
Relationship between breakdown pressure and interconnectivity number (ICN).

Figure 15 shows the relationship between the breakdown pressure and the interconnectivity number after fracturing for the five cases reported previously. There is a strong relationship between the ICN and fracture pressure. Lower breakdown pressure resulted in a very high interconnectivity number, and higher breakdown pressure yielded a very low interconnectivity number. This means in tight rocks with damage due to filter cake, fracturing itself may not be sufficient to have good production from the well, and additional operations such as filter cake and damage removal may be required to enhance the production and enhance the connectivity between the created fracture and the pore system in the rocks. This should be considered carefully in different rocks, such as tight and unconventional rocks.

The method proposed in this work can be used to evaluate the fracture treatment efficiency in creating and well-connected fractures to the pore system in the rock to have good communication between the fractures and the rocks and facilitate the production from tight reservoirs.

## 4. Breakdown Pressure Modeling

Experimental outcomes show that the filter cake’s existence, which acts as a seal, increases the breakdown pressure of the formation. From a theoretical perspective, the filter cake prevents any communication between the wellbore and the formation. The partial filter cake might not prevent communication, but it reduces the rock permeability around the wellbore. In this section, a model is created to show the impact of filter cake on the breakdown pressure. The model also is used to show how removing filter cake can reduce the breakdown pressure. It integrates a flow model (to simulate fluid injection) with popular analytical solutions to estimate the breakdown pressure. The breakdown pressure of the formation in a vertical wellbore assuming no fluid penetration can be estimated using the classical Hubbert and Willis [54] (H-W) approach as follows:

(2) $$p_{b}=3\sigma_{hmin}-\sigma_{Hmax}+T-p_{o}$$

where $p_{b}$ is the breakdown pressure, $\sigma_{hmin}$ is the minimum horizontal stress, $\sigma_{Hmax}$ is the maximum horizontal stress, $p_{o}$ is the pore pressure, and T is the formation tensile strength. The equation above can be used to predict the breakdown pressure assuming complete filter cake around the wellbore (no fluid penetration). For the cases where fluids penetrate the formation, the breakdown pressure declines with the increase in pore pressure. The breakdown pressure can be estimated using the Haimson and Fairhurst [55] (H-F) Equation as follows:

(3) $$p_{b}=\frac{3\sigma_{hmin}-\sigma_{Hmax}+T-2\eta p_{o}}{2\left(1-\eta \right)}$$

where

(4) $$\eta =\frac{\alpha \left(1-2\nu \right)}{2\left(1-v\right)}\;\;\;\;\;\;0\leq \eta \leq 0.5$$

and where α represents the Biot coefficient and ν is the Poisson ratio. Smith and Montgomery [56] stated that η=0.25 is suitable for field applications of breakdown prediction. Both H-W and H-F approaches assume that the breakdown will occur if the effective tangential stress is equal to the rock tensile strength. The equation above can be used to predict the breakdown pressure for no filter cake, partial filter cake, and complete removal of filter cake if the change in pore pressure is considered. The above equations do not consider the change in pore pressure as the fluid is injected. Detournay and Cheng [57] (D-C) investigated the impact of pressurizing rate on the breakdown pressure by providing the following analytical solution:

(5) $$p_{b}=\frac{3\sigma_{hmin}-\sigma_{Hmax}+T-2p_{o}}{1+\left(1-2\eta \right)h\left(\gamma \right)}+p_{o}$$

where $h\left(\gamma \right)$ is a function that represents the pressurizing rate. The advantage of the D-C model is that it returns the H-W model at a high pressurizing rate where no fluid is allowed to penetrate the formation. In this case, $h\left(\gamma \right)=0$, and hence, Equation (2) is obtained. It also returns Equation (3) under a slow pressurizing rate where fluid is allowed to penetrate the formation. In this case, $h\left(\gamma \right)=1$, and hence, Equation (3) is returned. Although the D-C model is incorporating the impact of pressurizing on the breakdown pressure, it is challenging to relate between the skin (i.e., reduction of permeability around the wellbore) and the pressurizing rate.

A flow model was incorporated in this study to estimate the increase in pore pressure as the fracturing fluid is injected. The skin factor or permeability reduction can be easily incorporated into the flow model. The mass conservation equation in porous media and Darcy’s law are used to estimate the pressure profile during injection as follows:

(6) $$\nabla \cdot \;\left(\;\rho u\right)=-\frac{\partial \left(\rho \phi \right)}{\partial t}$$

(7) $$u=-\frac{k}{\mu }\;.\nabla p$$

where ρ is the fluid density, u is the velocity vector, ϕ is the rock porosity, t is time, k is the permeability tensor, μ is the viscosity, and p is the pressure. The above equations were solved assuming no flow outer boundary conditions and constant flow at the wellbore location. The pore pressure is assumed to be equal to the average pressure surrounding the wellbore, $\bar{p}$, which is calculated as

(8) $$p_{o}=\bar{p}=\frac{\int pdV}{\int dV}$$

The model, which was coded using MATLAB, starts by solving the flow model to obtain both the wellbore pressure $p_{w}$ and the pore pressure $p_{o}$. The simulator stops when the breakdown occurs by satisfying the following criterion:

(9) $$p_{w}=p_{b}$$

The permeability around the wellbore (skin) is altered in the flow simulator to simulate different filter cake conditions. Equation (2) is used to estimate $p_{b}$ for complete filter cake, while Equation (3) is used for the other scenarios.

### Modeling Results

First, the classical H-W and H-F models can show the impact of complete filter cake on the breakdown pressure. As mentioned, the H-W model assumes no fluid penetration, which is suitable for predicting the breakdown pressure when a filter cake completely seals the formation. The H-F model can be used to estimate the breakdown pressure assuming no filter cake. Table 5 shows the input parameter to predict the breakdown pressure using the model above.

**Figure 16:**
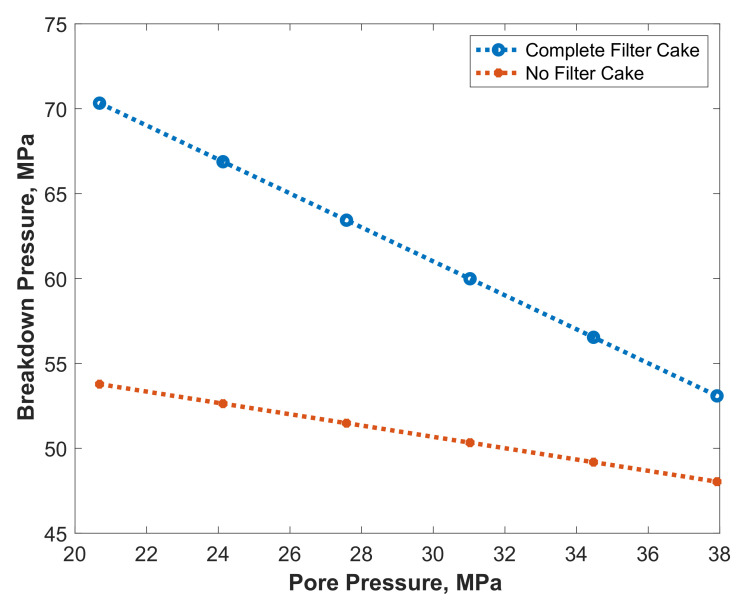
Impact of pore pressure on the breakdown pressure with and without filter cake.

Figure 16 shows the impact of filter cake on the breakdown pressure at different pore pressures representing different reservoir depths. It could be observed that the filter cake existence increases the breakdown pressure significantly, especially at low pore pressures. We used, in these simulations, constant *η* value; nevertheless, it might change with depth due to the change in Poisson ratio and Biot’s coefficient.

**Figure 17:**
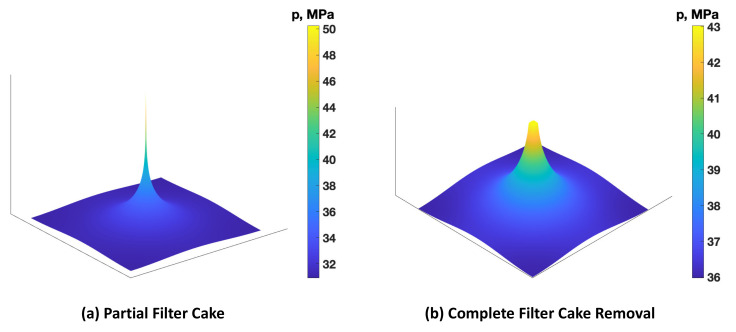
Pressure profile around the wellbore assuming (**a**) partial filter cake and (**b**) complete filter cake removal using TCF.

Using the coupled numerical flow and analytical breakdown pressure models, the breakdown pressure was predicted for partial filter cake and complete filter cake removal cases. For the partial filter cake case, the permeability around the wellbore was assumed to be reduced by 50%, compared to the original formation permeability. On the other hand, the permeability around the wellbore was assumed to increase to Darcy’s range (1 Darcy) due to TCF treatment. Additionally, due to the microcrack created by the TC, the formation tensile strength is reduced significantly (approaches zero). It is assumed that the damaged and stimulated zones are within 0.304 m from the wellbore. Figure 17 shows the outcomes of the flow model illustrating the pressurizing rate. Figure 17a shows that the pressure increases significantly in the wellbore due to the existence of filter cake. In contrast, Figure 17b shows that the pressure increase was lower in the wellbore, and the pore pressure surrounding the wellbore increased noticeably. According to Equation (3), high pore pressure would result in a lower breakdown pressure.

**Figure 18:**
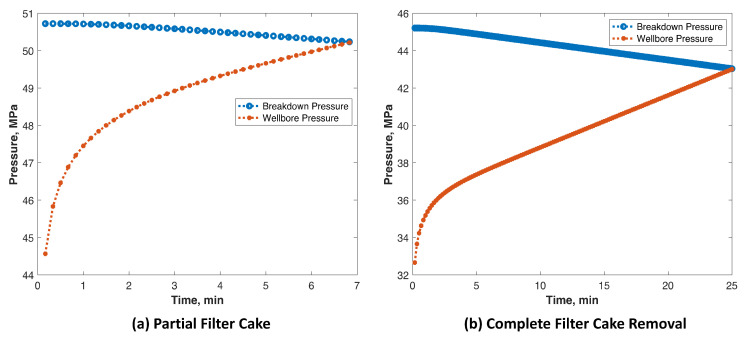
Prediction of the breakdown pressure using the integrated model assuming (**a**) partial filter cake and (**b**) complete filter cake removal using TCF.

Figure 18 shows that the formation breakdown occurs when the breakdown pressure is equal to the wellbore pressure. The breakdown pressure is not constant, and it changes with the increase in pore pressure, which increases with injection time. Additionally, the wellbore pressure increases with the injection time until reaching the breakdown pressure. The model shows that the breakdown pressure due to the existence of partial filter cake was 50 MPa. The TCF treatment reduced the breakdown pressure to 43 MPa due to increased pore pressure and reduced rock tensile strength. Obviously, it takes longer to achieve formation breakdown, assuming the same injection rate, after TCF treatments due to the high injectivity created. This allows a higher injection rate of fracturing fluids without creating high-pressure values at the surface. Fundamentally, increasing the injection rate should reduce the time at which formation breakdown occurs.

## 5. Conclusions

This study presented a novel approach to reduce the required breakdown pressure of the formation containing the water-based drilling fluid filter cake. Based on the experimental results, conducted analysis, and the discussion presented in this study, the following conclusions can be drawn:

1. Filter cake increased the breakdown pressure more than four times due to the wellbore strengthening effect. Breakdown pressure was increased from 6.9 MPa (case without filter cake) to 26.7 MPa (case with complete filter cake).
2. Partial removal of the filter cake with 20 wt% DTPA resulted in 40% removal of the filter cake. This resulted in a 33% reduction in breakdown pressure from 26.7 MPa (case with complete filter cake) to 17.9 MPa (case with partial filter cake).
3. The chelating agents such as 20 wt% DTPA, 10 wt% GLDA, and 9 wt% K_2_CO_3_ resulted in complete dissolution of the filter cake, and the required breakdown pressure of the rock was similar to the one without filter cake.
4. Using thermochemical treatment combined with DTPA to remove the filter cake resulted in a further decrease of the breakdown pressure due to the complete removal of the filter cake in addition to the creation of microfractures.
5. The compressive strength of the samples after fracturing experiments was aligned with the breakdown pressure.
6. The diffusion coupling through NMR scans confirmed the higher interconnectivity between different pore systems after the combined thermochemical and chelating agent treatment.
7. A strong relationship between the interconnectivity number (ICN) and fracture pressure was observed; lower breakdown pressure resulted in a very high interconnectivity number, and higher breakdown pressure yielded a very low interconnectivity number.
8. A model was created to predict the breakdown pressure at a field scale. It shows that filter cake removal in terms of TCF treatment can reduce the breakdown pressure significantly.

## Figures and Tables

**Table 1:** Different types of filter cake removal scenarios in oil- and water-based drilling fluids.

Filter Cake Type	Filter Cake Remover	Comments	References
Barite water-based mud	DTPA chelating agent + K_2_CO_3_ + Enzyme	Removal efficiency reached 90% at 270 °F	[21]
	EDTA chelating agent + K_2_CO_3_ + Enzyme	Removal efficiency reached 85% at 270 °F	[22]
	EDTA chelating + thermochemical	Removal efficiency reached more than 80% at different temperatures	[23]
Barite Oil-based mud	Multi-stage (Solvent + GLDA + K_2_CO_3_ + HCl)	Removal efficiency reached 83% at different temperatures	[24]
	EDTA chelating agent + K_2_CO_3_ + EGMBE	Removal efficiency reached 80% at different temperatures	[24]
Calcium carbonate water-based, drill in fluid	Acid precursor of organic acids	Filter cake removed efficiently	[25]
Barite filter cake	Formate brines	Barite solubility reached 3500 mg/L at 100 °C	[26]
Invert emulsion filter cake	Oil wetting agents, a precursor of organic acid	Uniform removal of filter cake	[27,28]
Invert emulsion filter cake	Microemulsion	Filter cake removal efficiency reached 97% after 24 h	[29]
Invert emulsion filter cake	Microemulsion	Efficient removal of the oil-based filter cake	[30,31]
Synthetic and oil-based drilling filter cake	Microemulsion + surfactants/co-surfactants	Efficient removal of the oil-based filter cake	[32]

**Table 2:** Petrophysical and rock mechanical properties of the tested samples.

Rock Parameters	Values	Units
Permeability	0.5	mD
Porosity	17	%
Unconfined Compressive Strength	41.3	MPa
Tensile Strength	8.9	MPa
Compressional Wave Velocity	3050	m/s
Shear Wave Velocity	1900	m/s
Dynamic Poisson’s Ratio	0.27	-
Dynamic Young’s Modulus	34	GPa
Bulk Density	2.7	g/cc

**Table 3:** Kinetics of thermochemical fluids.

Reaction Parameters	Values
C Jmol.K	85–110
λ Wm K	0.1–0.6
ΔHkJmol	369

**Table 4:** Experimental methodology of the breakdown pressure.

Experiments	Fracturing Fluid	Filter Cake Formation	NMR Scan	Scratch Test	Comment
1	Water	No	Yes	Yes	Base case experiment without filter cake.
2	Water	Yes	Yes	Yes	Breakdown pressure experiment with water on filter cake
3	20 wt% DTPA	Yes	Yes	Yes	Breakdown pressure experiment with DTPA chelating agent only on filter cake
4	20 wt% DTPA + 10 wt% GLDA + 9 wt% K_2_CO_3_	Yes	Yes	Yes	Breakdown pressure experiment with mixed chelating agent only on filter cake
5	Thermochemical + DTPA	Yes	Yes	Yes	Breakdown pressure experiment with chelating agent and thermochemical fluids only on filter cake

**Table 5:** Input parameters for the breakdown prediction model.

Input	SI Unit	Field Unit
k	9.869 × 10^−15^ m^2^	10 mD
ϕ	0.2	
Injection rate, q	0.053 m^3^/s	2 bpm
$\mathrm{Initial}\;\mathrm{pressure},\;p_{i}$	29.85 MPa	4330 psi
μ	1 mPa.s	1 cp
$\mathrm{Total}\;\mathrm{compressibility},\;c_{t}$	1.45 × 10^−4^ 1/MPa	1 × 10^−6^ 1/psi
$\sigma_{hmin}$	41.4 MPa	6000 psi
$\sigma_{Hmax}$	41.4 MPa	6000 psi
T	8.3 MPa	1200 psi
η	0.25	

## Data Availability

Not applicable.

## Sample Availability

Samples of the compounds are not available from the authors.
